# Surgery of the Liver and Bile Ducts

**Published:** 1904-07-30

**Authors:** 


### SURGERY OF THE LIVER AND BILE DUCTS.

Cholelithiasis.—Brewer[1] says that while it is generally recognised that jaundice and pain are the commonest, and often the only symptoms indicating the presence of a stone in the common duct, it is not as well recognised that a certain small number of these cases present in addition chills, fever, and sweating, so characteristically intermittent in character as strongly to suggest a malarial infection. Most modern observers regard the condition as one of septic absorption from infection of the duct—a true infective cholangitis. This view is strengthened by the fact that in severe and neglected cases, the ducts are thickened and hyperæmic, the liver the seat of multiple abscesses, and the bile changed in appearance by the presence of pus and blood. The condition is one of great gravity, and unless promptly relieved by surgical means, leads, almost invariably, to a fatal termination. The only treatment that can hold out any hope of success is choledochotomy, removal of the stones, and hepaticus drainage. This last consists in the introduction of a small rubber tube into the hepaticus, through the wound in the common duct. The wound is then partly closed by introducing two catgut sutures longitudinally on either side of the incision, and tying the opposite ends across the duct. A small strip of gauze is then placed over the closed wound, and secured by again tying the uncut ends of the catgut sutures over it. The free ends of the rubber tube and strips of gauze are brought out of the abdomen opening. If gall stones are removed also from the gall bladder, this is drained independently. The tube in the hepaticus is removed in about 10 days, and that in the gall bladder a little later. Lloyd[2] calls attention to the fact that gall-stone patients may continue to suffer from painful attacks, although all their gall stones have been passed or have been removed by operation; obstruction in the ducts—most often the cystic—depending on strictures which have been set up by the irritation of departed calculi. Post-operative biliary and mucous fistulæ are not infrequently due to this cause, and it is important, therefore, in every operation for the removal of gall stones from the gall bladder to assure oneself at the time of operation that there is no organic obstruction in the cystic duct beyond. Hartmann and Tuffier[3] alike advocate drainage in preference to suture of the common duct. Hartmann passes a big drainage tube between the liver and the transverse colon, placing its lower end against the incision in the duct. A strand of aseptic gauze is passed along each side of the tube. The gauze is removed on the fourth day, the drainage tube on the sixth. Salinari[4] recommends for calculous obstruction of the hepatic duct the digital fragmentation of the calculus whenever possible, if the fingers can be got beyond the stone so that the operator may be sure he is not pushing it back towards the liver.

Cyst of the Liver.—Doran[5] reports a case of large bile cyst of the liver, causing jaundice without cholelithiasis, and which was successfully treated by incision and drainage. He agrees with Kilvington that the curette should never be used; there is usually no epithelium lining big cysts that could secrete so as to fill the cyst again. That writer recommends that a simple cyst should be dropped back when all the fluid has been allowed to escape, but he adds that when calcareous plates are present or when there is any bile in the fluid drainage is necessary.

Resection of the Liver.—Anschütz[6] says that when it has been decided that the case is a proper one for resection the incision must be freely enlarged in any direction necessary, and the abdominal walls laid open, wide and gaping. The lowest ribs may require resection or the hepatic ligaments divided. Great care must be taken not to tear the liver, this risk being greater than is usually supposed. The fear of using the knife or scissors in cutting into the liver-tissue is an exaggerated one. Where the tumour is encapsulated it has been peeled out by the finger or a blunt instrument. The principal interest in liver resection attaches to the methods for the control of hæmorrhage. There is nothing in the nature of the case which forbids the application of ligatures to the normal hepatic vessels, but usually the surrounding tissues are so modified that this ideal method is impracticable. Of all the methods available the thermo-cautery is the favourite, and, used at a constant feeble red glow, it is certainly a pre-eminent styptic for the capillaries and small vessels, but with the larger it mostly fails. Tamponning may be employed either temporarily or as a permanent method of compression. Rowe[7] records a case in which he successfully excised a tuberculous mass from the liver.

Tuberculous Abscess of Liver.—Auvray[8] says primary hepatic tuberculosis is exceptional, but secondary is much commoner than is usually supposed. Perihepatitis is usually associated with it, resulting in adhesions to the diaphragm, abdominal wall and intestines. Perihepatic abscess gives rise to: (1) Various digestive disturbances—loss of appetite, nausea, vomiting, persistent diarrhœa—leading to rapid loss of flesh. (2) Rigors, with nocturnal elevations of temperature. (3) Pain of a dull, persistent character in the right hypochondrium, increased by pressure upon the lower ribs. (4) A swelling easily felt upon bimanual palpation. The evolution of the abscess is slow, and extends over several months. The prognosis is very grave, since, although the local lesion may be cured in some cases by surgical treatment, other manifestations of tubercle are usually present. The only rational treatment is free incision and drainage of the abscess and this is facilitated by previously resecting the costal margin.

[1] Med. Rec., Feb. 20, 1904, p. 296. [2] Birmingham Med. Rev., March 1904, p. 131. [3] Brit. Med. Jour., Dec. 5, 1903, p. 1480. [4] Brit. Med. Jour., Jan. 23, 1904, Epitome, No. 47. [5] Lancet, Oct. 31, 1903, p. 1218. [6] Med. Rec., Nov. 21, 1903, p. 819. [7] Annals of Surg., Jan. 1904, p. 98. [8] Med. Chron., Nov. 1903, p. 138.

